# Cutaneous perforator mapping and ink-based perfusion assessment in an ovine model: An anatomical study and systematic review

**DOI:** 10.1016/j.jpra.2026.06.014

**Published:** 2026-06-25

**Authors:** Ugo Lancien, Léa Passemard, François Thuau, Théodore Lahmar, Olivier Gauthier, Baptiste Charbonnier, Pierre Perrot

**Affiliations:** a Plastic, Reconstructive, and Aesthetic Surgery Unit, Nantes University Hospital, 1 place Alexis Ricordeau, 44000 Nantes, France; b INSERM, UMRS 1229, Laboratory Regenerative Medicine and Skeleton (RMeS), 1 place Alexis Ricordeau, 44000 Nantes, France; c Université de Nantes, UFR Odontologie, 1 place Alexis Ricordeau, 44000 Nantes, France; d ONIRIS Nantes – Atlantic College of Veterinary Medicine, Centre de Recherche et d’Investigation Préclinique (CRIP), Nantes, France

**Keywords:** Perforator flap, Anatomical study, Vascular mapping, India-ink perfusion, Ovine model, Microsurgery

## Abstract

**Background:**

A precise understanding of perforator anatomy and the corresponding cutaneous perfusion is essential for the design of perforator flaps in reconstructive surgery. Although large-animal models such as the sheep more closely reproduce human vascular dimensions than rodents, data integrating systematic anatomical mapping with functional perfusion assessment in this model are scarce.

**Methods:**

A systematic review (PRISMA 2020) was conducted in PubMed, Cochrane Central and Scopus to identify ovine studies investigating perforator anatomy and/or vascular injection techniques. Methodological quality was assessed with the MINORS tool. In parallel, an anatomical study was performed on seven adult ewes (*Ovis aries*). Five vessels were dissected on each side (10 vessels per animal, 70 perforators in total): the deep circumflex iliac (DCI), medial tarsal (MT), cranial tibial (CT), superior epigastric (SE) and thoracodorsal (TD). Perforator coordinates were recorded using a standardized orthogonal reference system based on reproducible anatomical landmarks. Pedicle length and vessel diameter were measured. A reproducible India-ink injection protocol was used to map the cutaneous territory perfused by each pedicle, quantified by digital planimetry (ImageJ).

**Results:**

The systematic review yielded eight heterogeneous studies, none of which combined perforator mapping with India-ink-based perfusion assessment in a sheep model (mean MINORS score 11.25 ± 3.01). The anatomical study identified five reproducible dominant pedicles with consistent spatial organization. Mean pedicle lengths ranged from 7.25 ± 1.57 cm (SE) to 17.43 ± 2.23 cm (TD). Mean perfused cutaneous areas ranged from 188 ± 36 cm² (CT) to 650 ± 84 cm² (DCI).

**Conclusion:**

This study provides the first combined anatomical and functional characterization of cutaneous perforators in the ovine model. The standardized mapping framework, quantitative vascular measurements and reproducible India-ink perfusion protocol provide a reproducible experimental framework for experimental research in perforator flap surgery and microsurgical training.

## Introduction

Perforator flaps have substantially advanced reconstructive surgery over the past four decades by allowing the transfer of well-vascularized skin flaps while sparing the underlying muscle and reducing donor-site morbidity. Their reliability depends on a detailed understanding of perforator anatomy and of the cutaneous territory supplied by each vessel.^1^^,^^2^

Experimental models have been instrumental in expanding our knowledge of perforator vascular anatomy and skin perfusion. However, small-animal models have intrinsic limitations regarding anatomical scale, vessel caliber and translational relevance.^3^ Large-animal models, and sheep in particular, offer more clinically relevant conditions, with tissue thickness and vascular dimensions suitable for experimental flap design and microsurgical procedures.

Despite these advantages, the literature on perforator anatomy in the ovine model remains scarce and fragmented. Existing studies are either limited to anatomical description or to vascular injection techniques, without integrating both approaches into a comprehensive vascular map. Moreover, the relationship between perforator anatomy and the actual perfused cutaneous territory has not been adequately characterized in this model.

The aim of the present work was therefore twofold: to systematically review the literature on perforator anatomy in the ovine model and to provide a comprehensive anatomical and functional mapping of cutaneous perforators in the sheep, including identification of the dominant pedicles and assessment of the corresponding cutaneous perfusion territories using a reproducible India-ink injection protocol.

## Materials and methods

### Systematic review

A systematic review was conducted in accordance with the PRISMA 2020 guidelines.^4^ PubMed, Cochrane Central and Scopus were searched from inception to January 2026, with no language restriction, using the following keyword combinations: “Ewe perforator flap”, “Ovine AND flap”, “Sheep AND perforator flap”, “Ovine model AND perforator flap”, “Perforator flap AND mapping AND ovine model”, “ovine model AND skin flap”, (“perforator flap” OR “cutaneous perforator”) AND (“skin perfusion” OR angiosome OR “vascular territory”) AND (anatomy OR dissection) AND (ovine model), and (“perforator flap” OR “microsurgery”) AND (“ovine model”) AND (reconstructive).

Two reviewers independently screened titles and abstracts and then assessed the full text of eligible studies; disagreements were resolved by consensus. Inclusion criteria were experimental studies in ovine models investigating perforator anatomy and/or vascular injection techniques. Exclusion criteria were human-only studies, case reports, conference abstracts and non-anatomical investigations.

Methodological quality was assessed with the MINORS (Methodological Index for Non-Randomized Studies) tool.^5^ MINORS was preferred over SYRCLE’s risk-of-bias tool^6^ because the included studies combined anatomical, *in vivo* and methodological designs that did not consistently fit the SYRCLE framework. Items are scored 0 (not reported), 1 (reported but inadequate) or 2 (reported and adequate); the maximum score is 16 for non-comparative studies and 24 for comparative studies.

### Anatomical study

The anatomical study was conducted on seven domestic ewes (*Ovis aries*), aged four to seven years and weighing on average 65 kg (range 57–78). The work was performed on the surgical platform of the National Veterinary School (ONIRIS, Nantes, France) in compliance with the requirements of the local Ethics Committee for Animal Experiments and reported in accordance with the ARRIVE 2.0 guidelines.^7^ Animals were euthanized by intravenous overdose of sodium pentobarbital (Euthasol, Dercha, France; 140 mg/kg). Immediately after euthanasia the sheep were weighed, fully shorn and prepared for dissection.

Five vessels per side were dissected and analyzed (10 vessels per animal, 70 vessels in total). The selected vessels were those most frequently reported in the literature: medial tarsal (MT), cranial tibial (CT), deep circumflex iliac (DCI), superior epigastric (SE) and thoracodorsal (TD) .^8^, ^9^, ^10^, ^11^ For each dissected vessel, the external caliber at its emergence from the source vessel and the length of the pedicle were measured with a micrometer ruler (Shinwa Rules Co. Ltd., Tsubame, Japan) using a previously published computer-assisted technique.^12^ The position of the origin of the pedicle and the position at which it reached the skin (site where the perforator pierces the deep fascia to enter the subcutaneous tissue, projected onto the overlying skin) were recorded on the cutaneous surface.

**Fig. 1:**
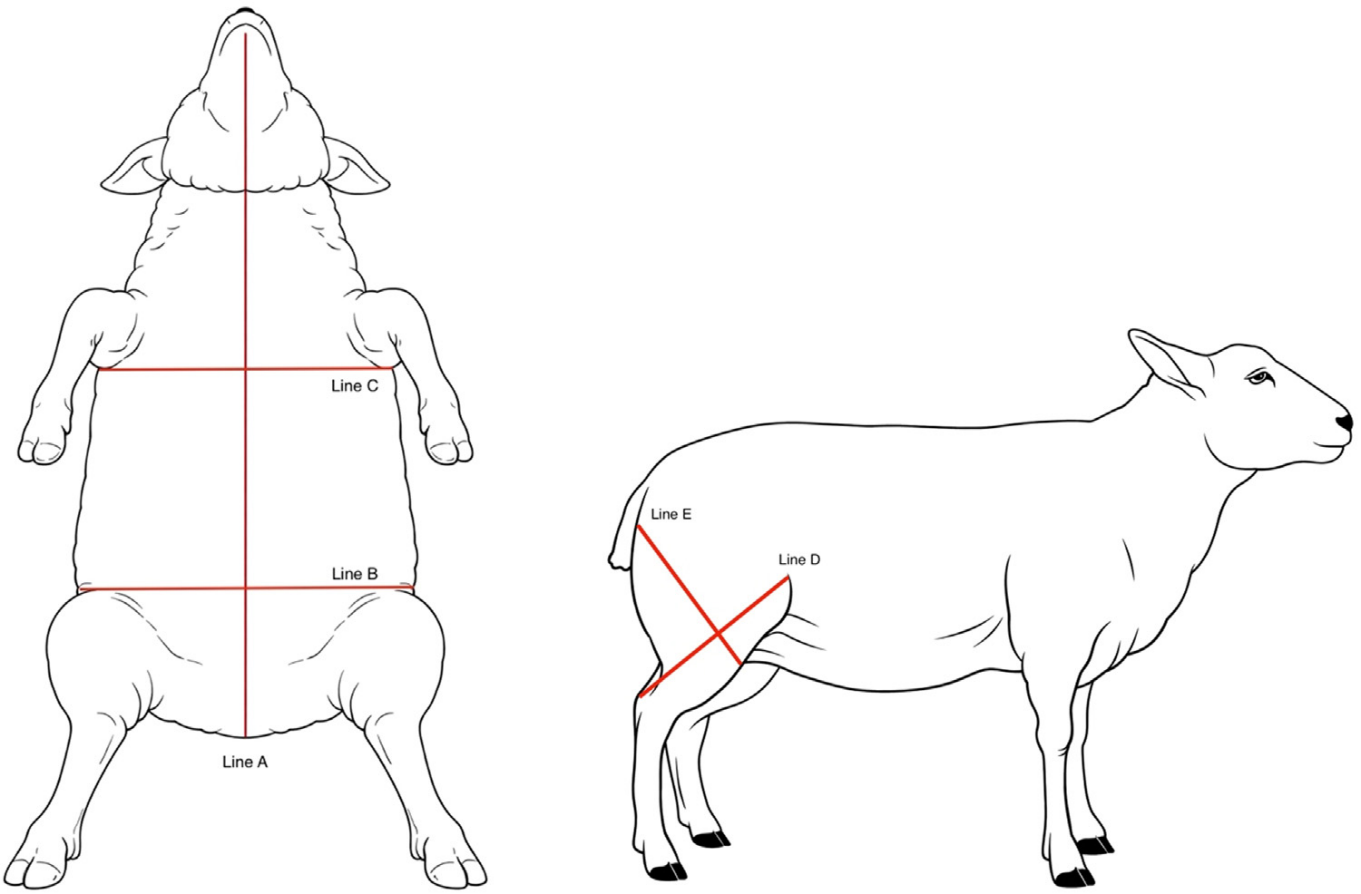
Diagram of the underside of a ewe showing the orthogonal reference axes used for perforator mapping. Line A: anatomical midline. Line B: line running along the top of the inguinal folds, perpendicular to line A. Line C: horizontal line passing through the xiphoid process and perpendicular to line A. Line D: line connecting the tuber coxae to the tarsal bone. Line E: line perpendicular to line D passing through its midpoint.

Two orthogonal reference systems were defined using anatomical landmarks (Fig. 1). With the animal supine: for the MT and DCI pedicles, the Y-axis ran along the anatomical midline (sternum, abdomen, mammary gland), and the X-axis was tangent to the iliac crest. For the CT pedicle, the Y-axis ran from the coxal tubercle to the tarsal bone and the X-axis intersected its midpoint perpendicularly. For the SE and TD pedicles, the Y-axis followed the anatomical midline and the X-axis was tangent to the xiphoid process. Coordinates were measured along these axes with a graduated ruler. Point clouds were generated for the origin and skin-arrival points; mean values are represented by crosses surrounded by ellipses delimiting the minimum-maximum range.

Each pedicle was selectively cannulated and a standardized intra-arterial injection of undiluted India ink was performed under controlled conditions: 2 mL of ink were injected over 5 seconds with a 3-mL syringe, after ligation of the pedicle proximal to the injection site. The protocol was adapted from Qassemyar et al.^13^ with the injected volume doubled to account for the larger body weight and vascular caliber of the ovine model. The extent of cutaneous staining was quantified by digital planimetry on standardized photographs using ImageJ (U.S. National Institutes of Health, Bethesda, MD, USA), with pixel-to-centimeter conversion based on a graduated ruler placed in the same plane as the vessel under study.

### Statistical analysis

Continuous variables are reported as mean ± standard deviation, with minimum and maximum values. Given the descriptive nature of the study, no inferential statistics were performed. Because no systematic left-right difference was expected or hypothesized, measurements from the right and left sides were pooled (*n* = 14 per pedicle: 7 ewes, both sides). Coordinates were expressed in a body-referenced system in which the X value represents the mediolateral distance from the anatomical midline and the Y value the craniocaudal position relative to the reference landmark, with a proximal-to-distal orientation for the limb pedicles (CT and MT). Right and left sides were therefore not analyzed separately. Analyses were performed using ImageJ (NIH, USA) and Microsoft Excel (Microsoft Corp., Redmond, WA, USA).

## Results

### Systematic review

**Fig. 2:**
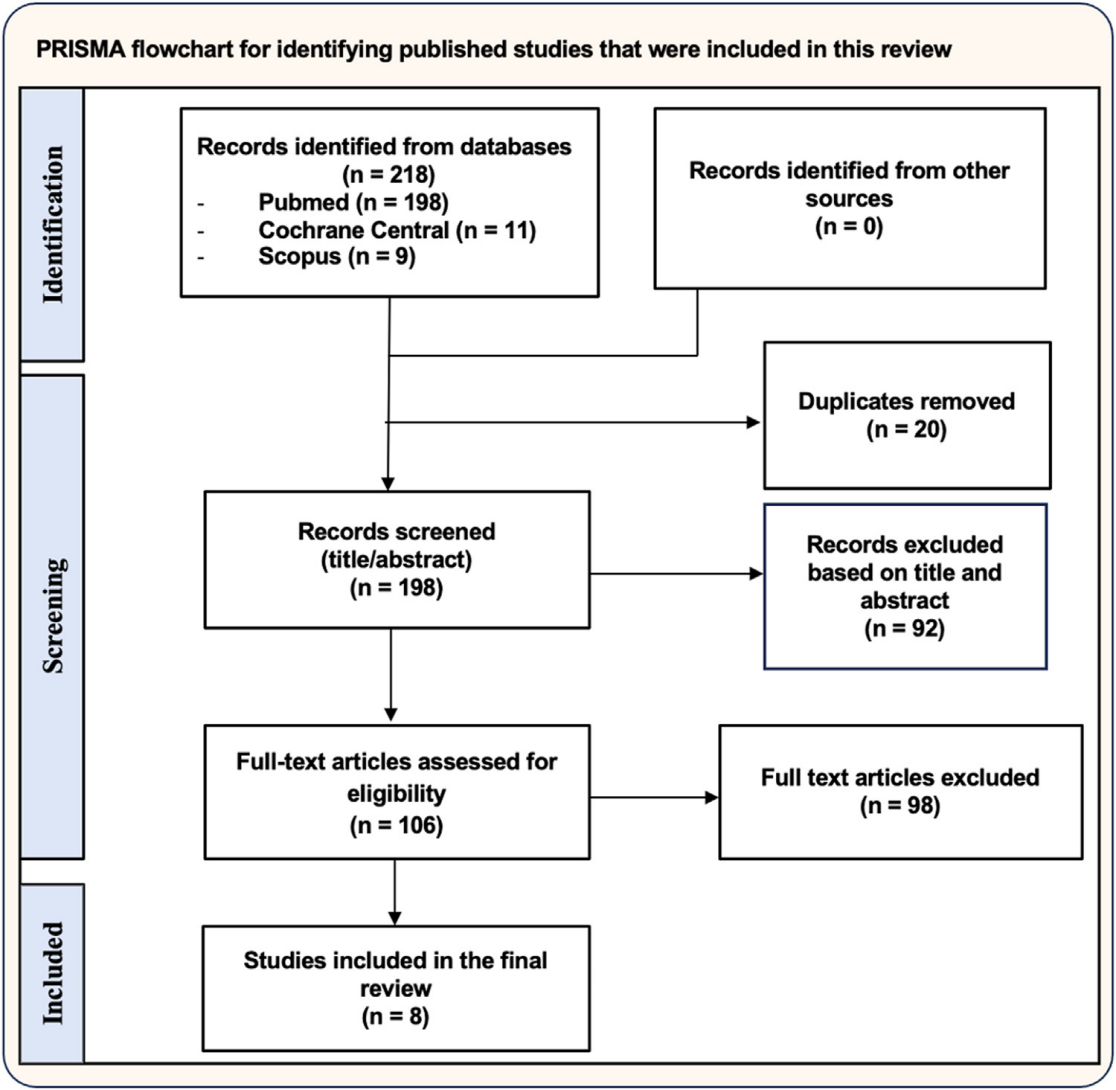
PRISMA 2020 flow diagram of the systematic review.

The initial search identified 218 records. After screening and eligibility assessment, eight studies were retained for inclusion. The PRISMA flowchart is presented in Fig. 2. The included studies were heterogeneous in terms of animal models, anatomical regions and methodologies. Two studies focused on anatomical dissection^14^^,^^15^, two on vascular injection techniques^11^^,^^14^ and five described pedicled skin flaps with perforator dissection.^8^, ^9^, ^10^^,^^16^^,^^17^ None of them combined systematic perforator mapping with functional assessment of cutaneous perfusion using India ink in a sheep model. The main characteristics of the included studies are summarized in Table 1. Across the included studies, the anatomical information available in the sheep concerns mainly the flank and the deep circumflex iliac territory, the abdominal wall, and the latissimus dorsi and thoracodorsal axis used for free or pedicled flaps; none provided a systematic perforator map or quantified the perfused cutaneous territory.

**Table 1:** Characteristics of the studies included in the systematic review.

Study	Method	Journal	Journal impact Factor (2024)	Vascular injection	Perforator mapping	Area of perfusion measure	Anatomical contribution
*In vivo* ovine flap model to evaluate surgical infection and tissue necrosis^11^	*In vivo* study	Journal of Surgical Research	1.7	Methylene blue and latex	No	No	Buttock pedicle flap on a branch of the external iliac artery (through the muscular septum); pedicle with artery, vein, nerve and efferent lymphatic vessel, plus musculocutaneous perforator to the distal watershed area.
The effect of venous obstruction in infected pedicle flap^17^	*In vivo* study	Archives plastic surgery	1.5	No	No	No	Buttock island pedicle flap on a cutaneous branch of the external iliac artery (artery, vein, nerve, efferent lymphatic); musculocutaneous perforator supplying the distal segment.
Effect of Ridogrel, a thromboxane receptor blocker and synthesis inhibitor on plasma and red blood cell flow in an arterial skin flap in sheep^8^	*In vivo* study	Scandinavian Journal Plastic of Reconstructive Surgery and Hand Surgery	0.9	No	No	No	Lateral abdominal arterial island flap on the inferior epigastric artery and concomitant veins, extended beyond its vascular territory (inferior epigastric axis)
A free flap system in sheep^15^	*In vivo* study	Microsurgery	1.7	No	No	No	Free flap system on the deep circumflex iliac artery (DCIA) of the flank: cutaneous (dorsal/lateral flank), myocutaneous (TFL perforators) and osteomyocutaneous (iliac crest segment) variants.
Anatomy of the blood supply to the flank of the sheep^14^	Anatomic study	Research in Veterinary Science	1.8	Arteriogram	No	No	Anatomy of the deep circumflex iliac artery (a. circumflexa ilium profunda) of the flank: dorsal flank branch, lateral flank branch, ventral/sub-stifle continuation; satellite veins (2.5–4 mm).
Evaluation of red blood cell and plasma flow and volume by the use of a dynamic and dual static acquisition radionuclide technique in arterial flaps in sheep^10^	*In vivo* study	Nuclear Medicine Communications	1.3	No	No	No	Lateral abdominal island flap on the inferior epigastric artery and concomitant veins, extended beyond its vascular territory (inferior epigastric axis).
Engineering Vascularized Transplantable Soft Tissue Free Flaps in Sheep Using the Arteriovenous Loop Technique^9^	*In vivo* study	Tissue Engineering Part A	2.9	No	No	No	Saphenous arteriovenous loop (saphenous artery-to-vein anastomosis) in the groin/medial thigh, embedded in Matriderm; femoral pedicle, free transfer to the neck (carotid artery, superior thyroid vein).
Radionuclide methodology for the assessment of the microcirculation in island pedicle flaps in a sheep model^16^	*In vivo* study	Nuclear Medicine Communications	1.3	No	No	No	Abdominal/inguinal island pedicle flap based on the inferior epigastric artery (3 mm) and concomitant veins; axial proximal part and random-pattern distal part with 4–5 perforators ligated.

The overall methodological quality of the included studies was moderate to low, with a mean MINORS score of 11.25 ± 3.01 (range 7–15) (Table 2). Common limitations were the absence of a prospective design with calculation of the study size, biased assessment of the study endpoint and an inappropriate follow-up period. Most of the included studies were published in the late 1980s or early 1990s, only one recent study (2025) reported the dissection of non-random flaps vascularized by perforator vessels.^9^

**Table 2:** Methodological quality of the included studies (MINORS scoring). Items are scored 0 (not reported), 1 (reported but inadequate) or 2 (reported and adequate). Maximum score: 16 for non-comparative studies. The references cited in this table are 8, 9, 10, 11^,^^14^, ^15^, ^16^, ^17^.

### Anatomical mapping

**Fig. 3:**
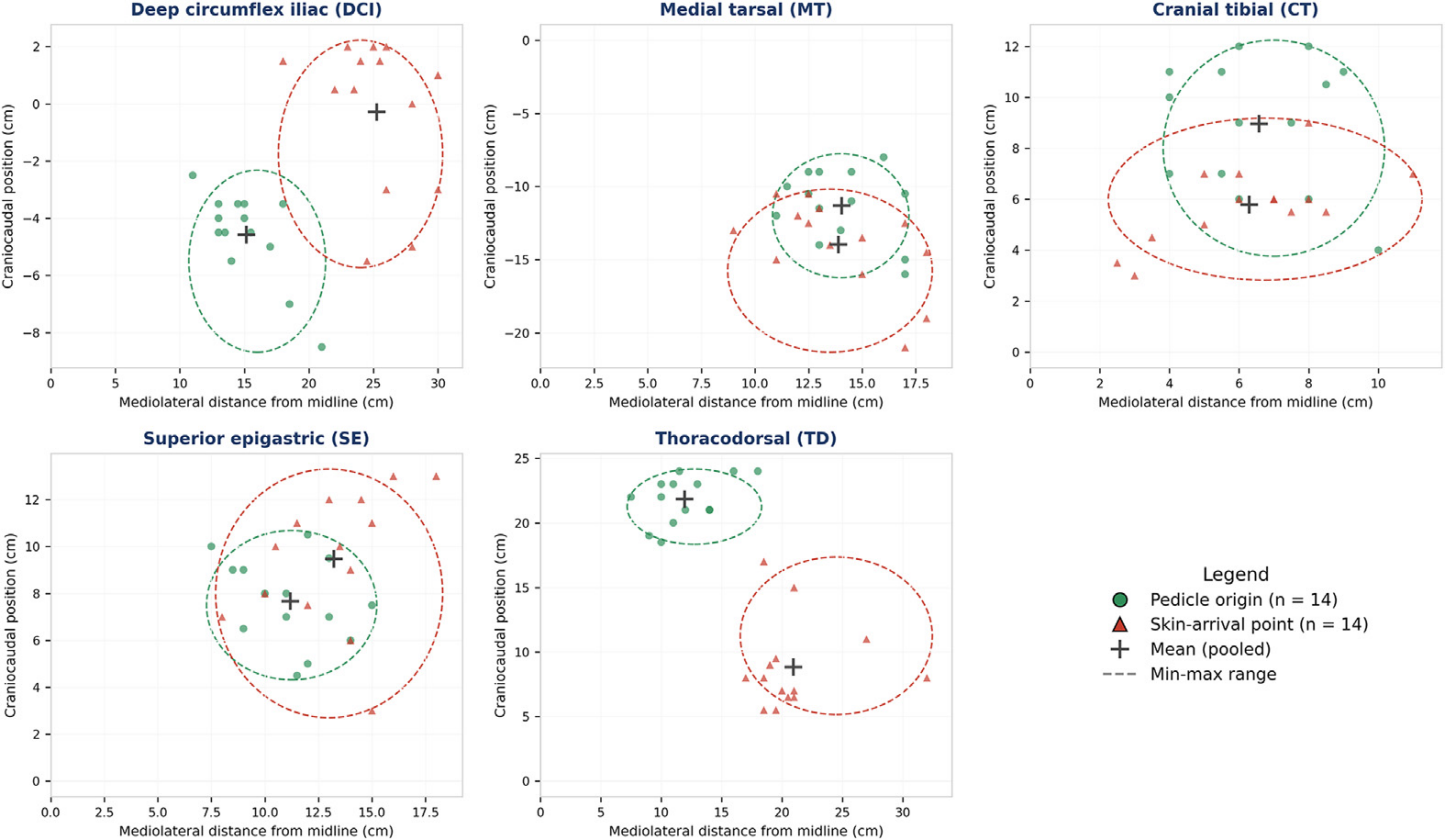
Pooled bilateral mapping of the cutaneous perforator coordinates of the five dominant pedicles (DCI, MT, CT, SE, TD), shown one panel per pedicle. For each pedicle, the origin points on the source vessel (green circles) and the skin-arrival points (red triangles) are plotted in a body-referenced system: the X-axis is the mediolateral distance from the anatomical midline, expressed as an absolute value so that right and left measurements combine, and the Y-axis is the craniocaudal position relative to the reference landmark. Measurements from both sides of all seven ewes are pooled (*n* = 14 per pedicle). The cross indicates the pooled mean and the dashed ellipse the minimum to maximum range. DCI: deep circumflex iliac; MT: medial tarsal; CT: cranial tibial; SE: superior epigastric; TD: thoracodorsal.

**Fig. 4:**
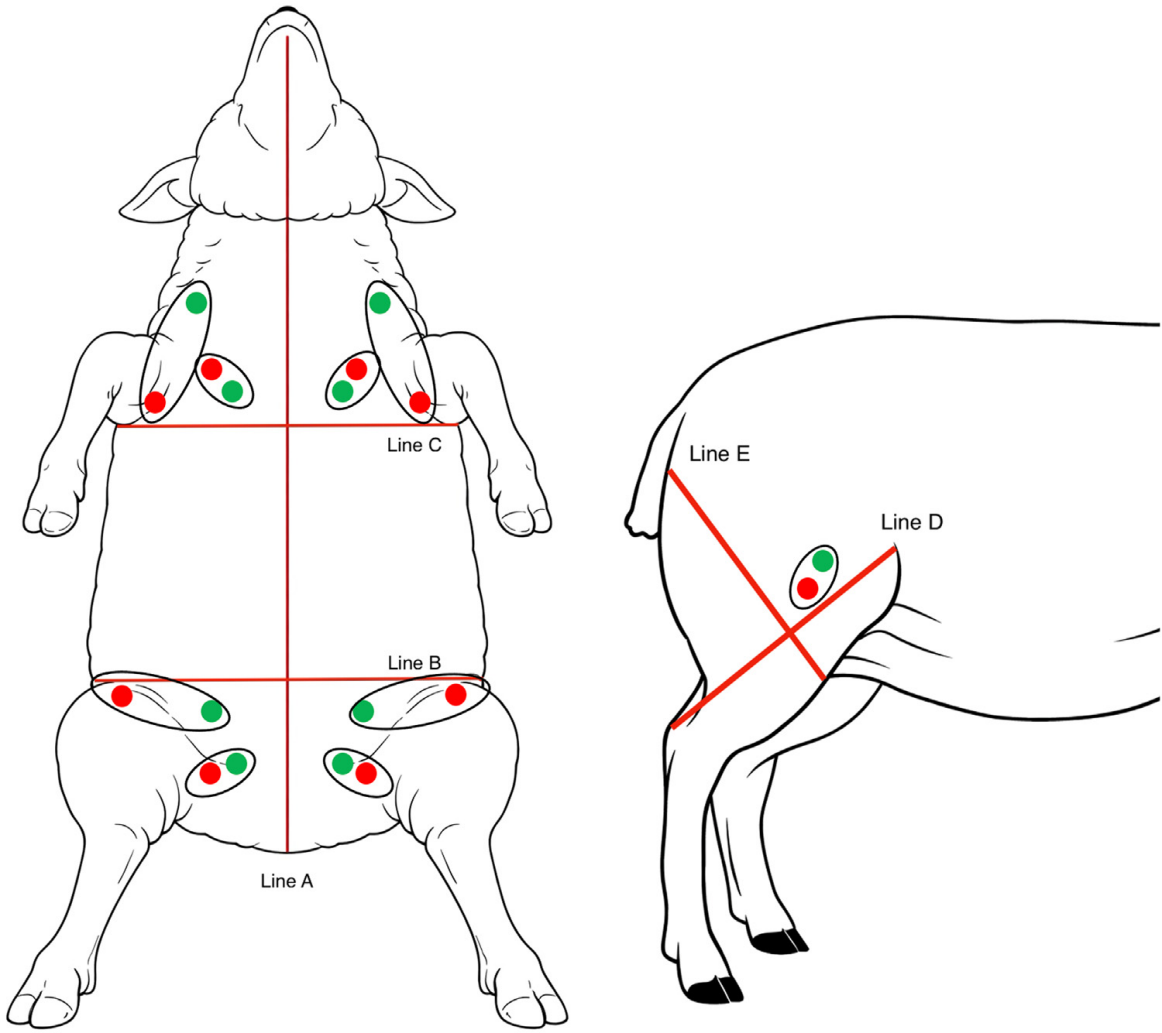
Synoptic atlas of the mean origin and skin-arrival points of the five dominant pedicles overlaid on the body of the ovine model. Green dots: origin points. Red dots: skin-arrival points.

Perforators were identified across the 70 predefined anatomical locations. Their distribution showed reproducible clustering patterns along specific anatomical axes. Scatter plots of the X and Y coordinates were generated for each vessel (Fig. 3). Mean coordinates of the origin on the source pedicle and of the skin-arrival point are reported below for each vessel. A synoptic atlas with mean origin and skin-arrival points overlaid on the body of the ovine model is provided in Fig. 4. Numerical results for the five pedicles are summarized in Table 3.

**Table 3:** Summary of the anatomical and perfusion data for the five dominant pedicles. Mean values ± SD. DCI: deep circumflex iliac; MT: medial tarsal; CT: cranial tibial; SE: superior epigastric; TD: thoracodorsal.

Pedicle	Origin (mediolateral; craniocaudal, cm)	Skin-arrival (mediolateral; craniocaudal, cm)	Pedicle length (cm)	Perfused area (cm²)	Artery diameter (mm)	Vein diameter (mm)
DCI	15.14 ± 2.67; −4.57 ± 1.57	25.25 ± 3.22; −0.29 ± 2.67	13.12 ± 1.72	650 ± 84	2.27 ± 0.40	2.77 ± 1.01
MT	14.04 ± 2.04; −11.32 ± 2.42	13.89 ± 2.84; −13.96 ± 3.03	10.39 ± 1.86	245 ± 66	1.48 ± 0.25	1.46 ± 0.33
CT	6.57 ± 1.95; 8.96 ± 2.55	6.29 ± 2.35; 5.79 ± 1.53	7.57 ± 2.68	188 ± 36	1.02 ± 0.34	1.33 ± 0.55
SE	11.18 ± 2.19; 7.68 ± 1.81	13.21 ± 2.62; 9.46 ± 2.90	7.25 ± 1.58	218 ± 67	1.29 ± 0.25	1.63 ± 0.33
TD	11.93 ± 2.83; 21.82 ± 1.81	20.93 ± 3.94; 8.82 ± 3.42	17.43 ± 2.23	461 ± 94	1.19 ± 0.21	2.69 ± 0.39

### Deep circumflex iliac (DCI)

May et al.^18^ and Zoltie et al.^14^ describe the deep circumflex iliac artery as a branch of the external iliac artery, arising approximately 5 cm from its origin. It gives off a cranial branch that supplies the abdominal muscles cranial to the coxal tubercle and reaches the cranial part of the thigh through the medial face of the tensor fasciae latae. It is accompanied by a satellite vein, a lymphatic vessel and the lateral cutaneous nerve of the thigh. In the present study, a 15-cm horizontal incision was made parallel to and below the line connecting the coxal tubercle to the udder. The thin subcutaneous tissue allowed deep dissection in an avascular plane between the abdomen and the lower limb, where the DCI pedicle was clearly visible at the bottom of the dissection space, with the vein running parallel to the artery. Pooling both sides (*n* = 14), the mean coordinates of the origin and of the skin-arrival point were (15.14 ± 2.67; −4.57 ± 1.57) and (25.25 ± 3.22; −0.29 ± 2.67), expressed as mediolateral distance from the midline and craniocaudal position. The mean pedicle length was 13.12 ± 1.72 cm (range 9.5–16). The mean arterial diameter at the origin was 2.27 ± 0.40 mm (1.49–2.87) and the mean venous diameter 2.77 ± 1.01 mm (1.91–5.30).

### Medial tarsal (MT)

May et al.^18^ describe the medial tarsal artery as the larger of the two terminal branches of the saphenous artery, dividing into medial and lateral plantar arteries that pass on either side of the calcaneal tubercle. The dissection started from the same incision as for the DCI and was extended distally in the subcutaneous plane. Pooling both sides (*n* = 14), the mean coordinates of the origin and of the skin-arrival point were (14.04 ± 2.04; −11.32 ± 2.42) and (13.89 ± 2.84; −13.96 ± 3.03), expressed as mediolateral distance from the midline and craniocaudal position. The mean pedicle length was 10.39 ± 1.86 cm.^9^, ^10^, ^11^, ^12^, ^13^, ^14^ The mean arterial diameter at the origin was 1.48 ± 0.25 mm (1.06–1.87) and the mean venous diameter 1.46 ± 0.33 mm (0.87–1.89).

### Cranial tibial (CT)

The cranial tibial artery is the larger branch of the popliteal artery; it passes between the fibrous part of the fibula and the tibia to reach the cranial surface of the tibia.^18^ The skin incision was made on the lateral aspect of the thigh in an inverted l-shape centered over the mid-thigh, and the dissection was carried out posteriorly in the subcutaneous plane. Pooling both sides (*n* = 14), the mean coordinates of the origin and of the skin-arrival point were (6.57 ± 1.95; 8.96 ± 2.55) and (6.29 ± 2.35; 5.79 ± 1.53), expressed as mediolateral distance from the midline and craniocaudal position. The mean pedicle length was 7.57 ± 2.68 cm.^4^, ^5^, ^6^, ^7^, ^8^, ^9^, ^10^, ^11^, ^12^, ^13^, ^14^ The mean arterial diameter at the origin was 1.02 ± 0.34 mm (0.40–1.78) and the mean venous diameter 1.33 ± 0.55 mm (0.76–2.54).

### Superior epigastric (SE)

The superior epigastric artery passes between the ninth costal cartilage and the xiphoid cartilage, then runs caudally on the abdominal surface of the rectus abdominis muscle, becomes embedded in it and supplies the ventral abdominal wall, anastomosing with the inferior epigastric artery.^14^^,^^18^ A vertical midline skin incision was made and lateral dissection was performed in the subcutaneous plane. Pooling both sides (*n* = 14), the mean coordinates of the origin and of the skin-arrival point were (11.18 ± 2.19; 7.68 ± 1.81) and (13.21 ± 2.62; 9.46 ± 2.90), expressed as mediolateral distance from the midline and craniocaudal position. The mean pedicle length was 7.25 ± 1.57 cm.^4^, ^5^, ^6^, ^7^, ^8^, ^9^, ^10^ The mean arterial diameter at the origin was 1.29 ± 0.25 mm (0.76–1.64) and the mean venous diameter 1.63 ± 0.33 mm (0.93–2.03).

### Thoracodorsal (TD)

The thoracodorsal artery is a branch of the subscapular artery; it crosses the medial face of the teres major muscle and runs dorsally and caudally along the surface of the latissimus dorsi muscle, supplying the latissimus dorsi, teres major, triceps brachii, caudal deep pectoral and cutaneous muscles, and the axillary lymph node.^15^^,^^18^ Pooling both sides (*n* = 14), the mean coordinates of the origin and of the skin-arrival point were (11.93 ± 2.83; 21.82 ± 1.81) and (20.93 ± 3.94; 8.82 ± 3.42), expressed as mediolateral distance from the midline and craniocaudal position. The mean pedicle length was 17.43 ± 2.23 cm (13.5–21). The mean arterial diameter at the origin was 1.19 ± 0.21 mm (0.87–1.67) and the mean venous diameter 2.69 ± 0.39 mm (1.87–3.22).

### Cutaneous perfusion

**Fig. 5:**
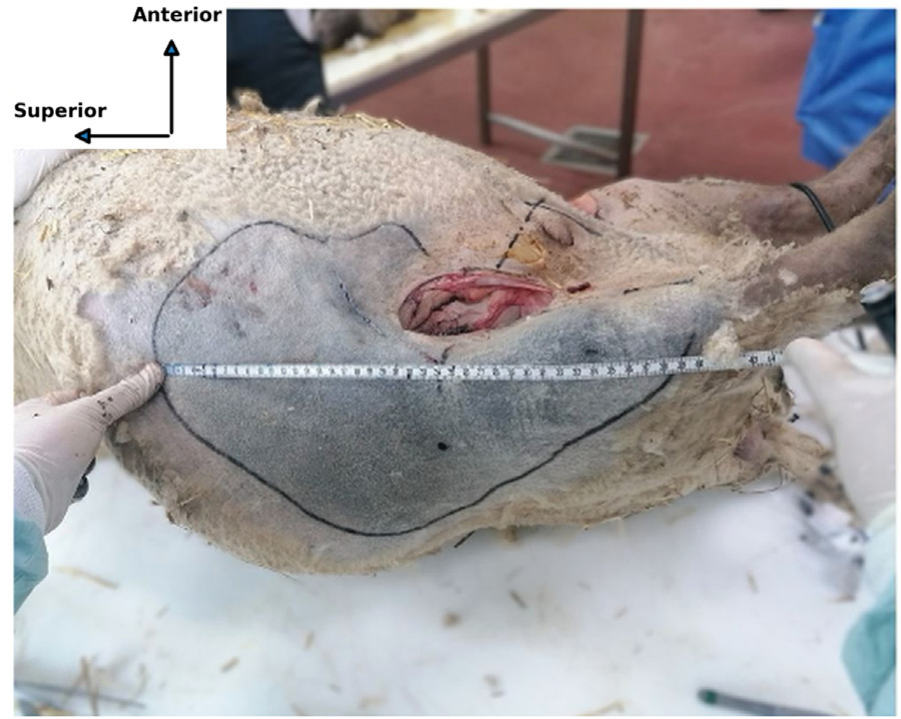
Representative cutaneous perfusion territory obtained after standardized intra-arterial India-ink injection, shown for the deep circumflex iliac (DCI) pedicle. The stained area was delineated and quantified by digital planimetry (ImageJ); the in-plane graduated ruler provides the pixel-to-centimeter scale. Perfused areas for all five pedicles are reported in Table 3.

India-ink injection produced clearly delineated cutaneous perfusion territories for each pedicle (Fig. 5). The mean perfused cutaneous areas were: DCI 650 ± 84 cm², MT 245 ± 66 cm², CT 188 ± 36 cm², SE 218 ± 67 cm² and TD 461 ± 94 cm². The deep circumflex iliac and thoracodorsal pedicles, which had the largest source calibres and the longest pedicles, also supplied the largest territories, whereas the cranial tibial and superior epigastric pedicles, with sub-millimetric to near-millimetric calibres, supplied the smallest, a pattern consistent with the perforasome concept. Quantitative analysis revealed substantial variability in the surface area supplied by each perforator, suggesting marked heterogeneity in vascular territories between pedicles.

## Discussion

The present study provides a combined anatomical and functional characterization of cutaneous perforators in the ovine model. The main findings are: a reproducible mapping of perforator origin and skin-arrival points based on a standardized orthogonal coordinate system; identification of five dominant pedicles (DCI, MT, CT, SE and TD) of interest for vascular and microsurgical research in the sheep; and quantitative assessment of cutaneous perfusion territories using a reproducible India-ink injection protocol.

A perforator atlas exists in human practice^19^ and is used to guide both clinical and experimental work by reporting the location of perforator vessels relative to anatomical landmarks. Inspired by this approach and by the wide clinical adoption of perforator flaps, we developed a similar atlas dedicated to the ovine model.

The choice of a large-animal model deserves a balanced appraisal. Among large animals, the pig is widely regarded as the reference species for cutaneous research, because porcine skin is histologically the closest to human skin in epidermal and dermal thickness, in its fixed relationship to the underlying fascia and in the organization of its direct and indirect cutaneous perforators; established porcine perforator and angiosome maps are therefore directly comparable to human data. The pig, however, carries significant practical drawbacks, including high acquisition and housing costs, the need for dedicated large-animal facilities, demanding handling and anesthesia, and substantial ethical and regulatory constraints. The sheep offers an intermediate profile: it is docile, less expensive, broadly available, and provides tissue thickness and vessel calibres within the range of clinical microsurgery, which makes it attractive for combined musculoskeletal, vascularized composite and bone tissue engineering work. The sheep should therefore be viewed as a pragmatic complement to, rather than a replacement for, the porcine model, particularly when the primary question concerns pure cutaneous perfusion.

Several species-specific features limit the direct extrapolation of ovine perfusion data to humans. In humans, the cutaneous vascular network is organized into perforasomes connected by direct and indirect linking vessels,^1^^,^^19^ with a comparatively reduced panniculus carnosus, so that skin perfusion depends predominantly on fasciocutaneous and musculocutaneous perforators. Quadrupeds such as the sheep retain an extensive cutaneous muscle layer that contributes both to skin mobility and to a segmental cutaneous blood supply, which may modify the size and linking behavior of adjacent vascular territories, and wool-bearing skin differs in dermal thickness and adnexal vascular demand. The territories reported here should therefore be interpreted as model-specific descriptors rather than as direct surrogates for human angiosomes.

The systematic review highlighted the paucity of data on perforator anatomy in the ovine model and confirmed the absence of studies integrating anatomical mapping with functional perfusion assessment in this species. Previous work either focused on descriptive anatomy or used vascular perfusion in isolation, with methylene blue and latex to assess flap necrosis^11^, or with arteriography of the ewe flank without specification of the contrast agent^14^, without ever establishing a direct correlation between perforator characteristics and the resulting perfused skin territory. The included studies showed substantial methodological heterogeneity (animal models, study designs, anatomical regions, vascular assessment techniques), which prevents direct comparison and limits the generalizability of their findings. The overall methodological quality, as measured by MINORS, was moderate to low, with recurrent limitations including absence of a prospective design, lack of standardized measurement protocols and potential bias in endpoint assessment. The review has several limitations. The number of eligible studies was small and they were markedly heterogeneous in model, anatomical region, design and vascular-assessment technique, which precluded any quantitative synthesis. Most reports predate the modern perforator concept and were not designed to map perforator anatomy, so the anatomical information they provide is fragmentary. The broad inclusion criteria, although necessary given the scarcity of data, mean that the review reflects the wider ovine flap literature rather than ovine perforator anatomy in the strict sense. Restriction to three databases and to indexed publications may have introduced selection and publication bias, and the MINORS instrument, although suited to non-randomized studies, was not specifically designed for anatomical work.

Although most of the identified studies date from the 1980s and 1990s, reflecting experimental approaches that did not yet incorporate the modern concepts of perforator mapping or quantitative perfusion analysis, research on perforator vessels is currently expanding rapidly and requires a precise knowledge of vessel locations to design reproducible experiments. Vascular diameters and pedicle lengths are also critical to assess the feasibility of microsurgical reconstruction and free-flap procedures. In this context, the present work fills a significant gap by combining both approaches and by providing the corresponding quantitative data.

Several methodological aspects strengthen the validity of this study. First, the use of a standardized orthogonal coordinate system based on anatomical landmarks allows reproducible mapping of perforator locations, which is essential for both experimental reproducibility and potential surgical translation. Second, quantitative measurements of pedicle length and vessel diameter provide the objective data that are usually lacking in anatomical studies and allow a hierarchy of vascular pedicles to be defined. Third, India-ink injection is a simple, reproducible and inexpensive method to assess cutaneous perfusion. Unlike more complex imaging techniques, it allows direct macroscopic visualization of the perforator territory.

From a microsurgical standpoint, the five pedicles differ markedly in their suitability for transfer. The deep circumflex iliac and thoracodorsal pedicles combined the largest calibres, the longest pedicles and the largest perfused territories, which makes them the most promising donor sites for experimental free-flap and large-territory work in the sheep. The cranial tibial and superior epigastric pedicles, with smaller calibres and territories, are better suited to supermicrosurgical training or to perforator-based local flaps. Although the descriptive design precludes formal correlation analysis, larger source calibres were broadly associated with larger stained territories, providing a rational basis for donor-site selection in future functional studies.

India-ink injection has intrinsic limitations: it does not reproduce pulsatile flow, vascular tone or active microcirculatory regulation, and the absence of a dynamic component means that the stained areas reflect the maximal anatomical filling territory rather than the physiological perfusion territory of a living flap. Passive diffusion of the dye beyond the true vascular boundary may overestimate the territory, and the extent of staining depends on injection volume, pressure and rate, which we sought to standardize through a uniform protocol but could not eliminate as a source of variability. These measurements should accordingly be read as static, worst-case anatomical estimates. The study was deliberately restricted to perforators of the ventral surface, because the ovine model, being a ruminant, cannot be maintained prone for extended periods during *in vivo* procedures, which makes the dorsal surface less directly relevant to clinical translation. Cadaveric specimens may underestimate vessel caliber because of incomplete vascular filling; our analysis therefore reflects a “worst-case” scenario, providing pragmatic estimates that can guide future investigations. Several techniques can assess cutaneous perfusion without dye injection: indocyanine green fluorescence angiography provides dynamic, real-time mapping of skin perfusion *in vivo*; computed tomographic and magnetic resonance angiography depict perforator course and caliber three-dimensionally; laser Doppler flowmetry and laser speckle contrast imaging quantify microcirculatory flow; dynamic infrared thermography maps perfusion through surface temperature; and microsphere techniques allow regional flow quantification. Compared with these methods, India-ink injection is static and qualitative, but it remains simple, inexpensive and well suited to *ex vivo* territory delineation, and the dynamic techniques above represent the logical next step for *in vivo* validation. cBeyond perforator-flap research, the ovine model has direct relevance to other translational applications, including the engineering of vascularized composite tissue constructs and bone tissue engineering using axial vascular pedicles, where reliable knowledge of donor-site perforator anatomy is a prerequisite. The present anatomical mapping is a first step that requires functional validation. This validation is planned in a live ovine model, combining flap elevation and flap-survival assessment with dynamic perfusion imaging (indocyanine green fluorescence angiography and laser Doppler or laser speckle flowmetry) and with correlation between source-vessel caliber and the viable skin territory. Only such studies will establish whether the dominant pedicles identified here translate into reliable experimental flaps.

## Conclusion

This study provides a standardized anatomical and descriptive characterization of cutaneous perforators in the ovine model, combining reproducible mapping, quantitative vascular measurements and a static India-ink perfusion assessment. These data offer a foundational anatomical framework that may support the design of future experimental flap studies in the sheep. They remain descriptive and require functional validation, through flap elevation, flap-survival analysis and dynamic perfusion imaging, before any conclusion can be drawn regarding flap viability or direct clinical translation.

## CRediT author statement

Ugo Lancien: Conceptualization, Methodology, Investigation, Writing - original draft. Léa Passemard: Conceptualisation, Investigation, Writing - original draft. François Thuau: Conceptualisation, Validation. Théodore Lahmar: Writing - review & editing. Olivier Gauthier: Supervision, Writing - review & editing. Baptiste Charbonnier: Supervision, Writing - review & editing. Pierre Perrot: Supervision, Writing - review & editing.

## Ethics approval

All experimental procedures complied with the requirements of the local Ethics Committee for Animal Experiments at our university and with the ARRIVE 2.0 guidelines.

## Funding

This research received no external funding.

## Declaration of competing interest

The authors declare that they have no known competing financial interests or personal relationships that could have appeared to influence the work reported in this paper.
